# Temporal evaluation of efficacy and quality of tissue repair upon laser‐activated sealing

**DOI:** 10.1002/btm2.10412

**Published:** 2022-09-28

**Authors:** Deepanjan Ghosh, Christopher M. Salinas, Shubham Pallod, Jordan Roberts, Inder Raj S. Makin, Jordan R. Yaron, Russell S. Witte, Kaushal Rege

**Affiliations:** ^1^ Biological Design Graduate Program, School for Engineering of Matter, Transport, and Energy Arizona State University Tempe Arizona USA; ^2^ James C. Wyant College of Optical Sciences University of Arizona Tucson Arizona USA; ^3^ School of Life Sciences Arizona State University Tempe Arizona USA; ^4^ School of Osteopathic Medicine A.T. Still University Mesa Arizona USA; ^5^ Department of Chemical Engineering, School for Engineering of Matter, Transport, and Energy Arizona State University Tempe Arizona USA; ^6^ Department of Medical Imaging University of Arizona Tucson Arizona USA

**Keywords:** incisional wounds, laser‐activated sealing, photoacoustic imaging, skin barrier function recovery, tissue adhesive, tissue repair, ultrasound

## Abstract

Injuries caused by surgical incisions or traumatic lacerations compromise the structural and functional integrity of skin. Immediate approximation and robust repair of skin are critical to minimize occurrences of dehiscence and infection that can lead to impaired healing and further complication. Light‐activated skin sealing has emerged as an alternative to sutures, staples, and superficial adhesives, which do not integrate with tissues and are prone to scarring and infection. Here, we evaluate both shorter‐ and longer‐term efficacy of tissue repair response following laser‐activated sealing of full‐thickness skin incisions in immunocompetent mice and compare them to the efficacy seen with sutures. Laser‐activated sealants (LASEs) in which, indocyanine green was embedded within silk fibroin films, were used to form viscous pastes and applied over wound edges. A hand‐held, near‐infrared laser was applied over the incision, and conversion of the light energy to heat by the LASE facilitated rapid photothermal sealing of the wound in approximately 1 min. Tissue repair with LASEs was evaluated using functional recovery (transepidermal water loss), biomechanical recovery (tensile strength), tissue visualization (ultrasound [US] and photoacoustic imaging [PAI]), and histology, and compared with that seen in sutures. Our studies indicate that LASEs promoted earlier recovery of barrier and mechanical function of healed skin compared to suture‐closed incisions. Visualization of sealed skin using US and PAI indicated integration of the LASE with the tissue. Histological analyses of LASE‐sealed skin sections showed reduced neutrophil and increased proresolution macrophages on Days 2 and 7 postclosure of incisions, without an increase in scarring or fibrosis. Together, our studies show that simple fabrication and application methods combined with rapid sealing of wound edges with improved histological outcomes make LASE a promising alternative for management of incisional wounds and lacerations.

## 1 INTRODUCTION

Soft tissue trauma, including lacerations and surgical incisions, require effective and rapid closure in order to minimize blood loss, prevent infection and promote healing. Surgical sutures and staples are the most commonly used devices for approximating soft tissue trauma including in the skin.^1^, ^2^ Although effective in superficial layers of skin, sutures do not integrate with the tissue, do not lead to immediate closure, and generally do not demonstrate optimal performance in deeper layers of the tissue, including in the hypodermis. In addition, tissue strength is suboptimal at early times after tissue approximation, which, by itself and in case of infections, can compromise effective healing.

Localized conversion of laser light energy to heat energy using endogenous or exogenous chromophores^3^ results in rapid photothermal sealing of soft tissues.^3^, ^4^, ^5^, ^6^, ^7^, ^8^, ^9^, ^10^, ^11^ Laser‐activated sealants (LASEs) in which exogenous chromophores are incorporated within a biomaterial sealant matrix offer promising alternatives to sutures and staples. In this approach, laser irradiation of the LASE‐tissue interface and the concomitant photothermal response can facilitate interdigitation of LASE biomolecules and tissue proteins, which results in rapid sealing and effective repair of soft tissues. We have previously reported the fabrication and characterization of LASEs as an approach for the rapid sealing and repair of ruptured tissues.^11^, ^12^, ^13^, ^14^, ^15^ The LASE system comprises of three components: (i) a matrix consisting of biomaterials, such as elastin‐like polypeptides, collagen, or silk fibroin, which integrate with the tissue upon sealing and act as a scaffold for aiding repair, (ii) chromophores including gold nanorods (GNRs) or the FDA‐approved dye, indocyanine green (ICG), which convert laser light energy to heat energy (photothermal energy conversion), thus resulting in a local increase in temperature, and (iii) a hand‐held near‐infrared (NIR) laser tuned to 808 nm that is used to carry out the tissue sealing procedure using LASEs. The rapid bonding of wound edges mediated by interdigitation of tissue proteins, leading to rapid sealing, has been demonstrated for temperatures ranging from 50–60°C.^16^, ^17^ A recent report which investigated the effect of temperature on tissue sealing observed highest welding strengths of tissue at 55°C. Use of elevated temperatures greater than or equal to 65°C led to denaturation of tissue proteins and negatively impacted tissue tensile strength.^18^ Our previous results have shown that LASE‐mediated tissue sealing results in improved recovery of tissue biomechanical properties in live mice, compared to Vetbond, a cyanoacrylate‐based skin glue.^11^, ^14^ In addition to facilitating sealing and repair, LASEs can be loaded with antibacterial drugs in order to combat methicillin‐resistant *Staphylococcus aureus* infection in at surgical site, thus protecting the tissue.^15^

ICG is an FDA‐approved dye that absorbs and emits light in the NIR region of the wavelength spectrum. Upon irradiation with NIR lasers, approximately 85% of the energy absorbed by the dye is converted into heat, which makes ICG a good photoconverter for various applications including photothermal sealing and photodynamic therapy.^19^, ^20^ In addition, ICG dye has a relatively short clearance period of 60–80 min from the body and is excreted unchanged via bile.^21^ The biodistribution and toxicity profiles of ICG dye are better understood compared to that for nanoparticles that are used as chromophores. It may also be possible to minimize batch‐to‐batch variation in LASE properties and performance by using the well‐established ICG dye.

In this study, we carried out a detailed investigation into the efficacy of laser tissue sealing using functional, biomechanical, visual (imaging), and histological evaluation at different time points during the course of healing following surgery, and compared these outcomes to those seen with sutures. ICG dye‐loaded silk fibroin (“silk”) films were used for sealing 1 cm, full‐thickness incisional wounds in BALB/c immunocompetent mice and transepidermal water loss (TEWL), and ultimate tensile strength (UTS) of skin were determined in order to investigate functional and biomechanical recovery, respectively, following tissue approximation. A combination of ultrasound (US) and photoacoustic imaging (PAI) along with histological evaluation was carried out in order to further visualize and gain insights into LASE‐mediated tissue repair.^22^, ^23^, ^24^, ^25^, ^26^, ^27^ These findings indicate that LASE‐mediated tissue sealing is significantly more effective at restoring function and biomechanical properties of skin compared to sutures at early time points following surgery.

## 2 EXPERIMENTAL

### 2.1 Materials

Silkworm (*Bombyx mori*) cocoons were purchased from Mulberry Farms as a source of silk fibroin protein (henceforth referred to as silk). Sodium carbonate (Na_2_CO_3_), and lithium bromide (LiBr) were purchased from Millipore Sigma for silk fibroin extraction from silkworm cocoons. Dialysis bags, 3.5 kDa molecular weight cut‐off (Spectra/Por), were purchased from Fisher Scientific to facilitate purification of silk fibroin. ICG dye was purchased from MP Biomedicals (#ICN15502050) and stored at 4°C. All solutions were freshly prepared in nanopure water (NPW; resistivity ~18.2 MΩ cm; Millipore Filtration System). BALB/c mice were purchased at ~10 weeks from Charles River Laboratories. Commercially available 4‐0 Monosof™ Monofilament Nylon Sutures (Medtronic) were purchased from esutures.com.

### 2.2 LASE fabrication

Silk fibroin was extracted from *B. mori* silkworm cocoons using previously described protocols.^14^, ^28^ Briefly, silkworm cocoons were degummed in a boiling 0.02 M Na_2_CO_3_ (Sigma‐Aldrich) solution for 30 min, washed in NPW three times, and dried at room temperature (RT). Degummed silk fibroin was dissolved in 9.3 M LiBr solution at 60°C for 4 h, centrifuged to separate insoluble contents, and dialyzed for 72 h at 4°C against a 3.5 kDa membrane in order to remove LiBr and impurities. Dissolved silk fibroin solution was centrifuged at 14,000 rpm for 20 min to remove remaining impurities. Stock ICG solution (5 mM dissolved in NPW) was added to aqueous silk fibroin solution (6 wt%) and homogenously mixed to obtain a final ICG concentration of 0.1 mM. This solution (500 ml) was poured over 2 cm × 2 cm square plastic coverslips and dried overnight at RT to obtain silk‐ICG LASE films or simply LASE films. The LASE films generated using this method had approximately 0.31 mg per film and all films were stored at RT prior to further use.

### 2.3 LASE characterization

Absorbance spectra of ICG solution (0.1 mM), as‐prepared LASE, LASE dissolved in saline to form a viscous paste, and LASE after laser irradiation were recorded from 400 to 999 nm using UV–Vis absorption spectroscopy (Synergy 2 Multi‐Mode Reader; BioTek Instruments). Absorbance spectra of NPW and silk fibroin film (LASE without ICG) were also recorded as controls for ICG solution and LASE, respectively. A hand‐held, continuous wave NIR laser (LRD‐0808; Laserglow Technologies), coupled with armored optical fiber with FC/PC connector (#AFF2001X, 1 m in length, and 200 μm in core diameter), and tuned to 808 nm was used for laser irradiation. The fixed laser spot size was 2 mm. A FieldMate laser power meter was used to measure the power of the laser beam, and power density of the laser beam was calculated by dividing the power of the laser beam by the area of the beam. An A325sc infrared (IR) camera (FLIR), equipped with a 10 mm 45° lens, was used to determine the surface temperature of LASE during laser exposure.

### 2.4 Sealing of full‐thickness incisional wounds in mice

BALB/c mice (10–12 weeks, weighing ~22–25 g; Charles River Laboratories) were used in this study and were housed in groups of five until surgery. All animal care and procedures were performed in strict compliance with protocols approved by the Institutional Animal Care and Use Committee (IACUC) at Arizona State University. Before surgery, mice were anesthetized with 120 mg/kg ketamine and 6 mg/kg xylazine (100 μl cocktail) by intraperitoneal injection. Dorsal hair was clipped, and the skin was prepped using three alternating swabs of chlorhexidine gluconate and 70% isopropyl alcohol. Two 1‐cm full‐thickness incisions were made side‐by‐side on the back of each mouse spaced roughly 2 cm apart using sterile scalpel blades (#15; Integra Miltex).^29^ In case of suture‐closed incisions, four evenly spaced simple interrupted knots were used to close a 1‐cm incision using 4‐0 nylon suture (#SN5699G; Medtronic; Monosof Black 18″ P‐13 cutting). For LASE‐sealed incisions, 10 μl of phosphate‐buffered saline (PBS; pH 7.4) were topically applied and a 1.2 cm × 0.5 cm LASE film was placed over the incision; contact of LASE with PBS resulted in quick dissolution of the film to form a viscous paste between the incision edges. The incision edges were approximated using a forceps, and the incised edges were aligned prior to laser sealing. The LASE‐tissue interface (incision line) was irradiated at a rate of 0.5 mm/s with the NIR laser tuned to 808 nm (CW) for 1 min while keeping the incision line approximated using forceps. The laser was applied at an angle between 60° and 80° to the skin at a power density of ~5.1 W/cm^2^ (~160 mW power output, ~2 mm laser beam diameter), corresponding to temperatures in the range of 50–60°C at the skin−LASE surface.^15^ Closure of left and right incisions with sutures or LASE were randomized. The mice were allowed to recover on heating pads until mobile and were housed individually. Incisions were assessed every day for up to 7 days postsurgery for any signs of infection, suture removal, or wound dehiscence and mice with any of these conditions were removed from the study.

### 2.5 Measurement of TEWL of healing wounds

TEWL is a measurement of change of water vapor density across the stratum corneum layer and is used to assess the barrier function of skin. Disruption to skin due to trauma, injury, wounds results in elevated TEWL levels and is indicative of weaker barrier function.^30^, ^31^ In this study, TEWL was measured on Days 2, 4, 7 postsurgery using a portable, closed chamber VapoMeter device (#SWL5580; Delfin Technologies). The VapoMeter was fitted with a small adapter (4.5 mm diameter, ~16 mm^2^ surface area) and a closed chamber was created on skin contact during the duration of measurement (~9–15 s). Ambient relative humidity and temperature (°C) were recorded during every measurement using a room sensor (#RHD1367) supplied along with the VapoMeter. For every TEWL reading, three consecutive readings were acquired along nonoverlapping regions over an incision area and the chamber was passively ventilated between every measurement.^32^, ^33^ TEWL readings of unwounded skin of sham mice were acquired on the same days. In all cases, TEWL values were recorded using the DMC software (Delfin Technologies) and values are displayed as mean ± standard error of mean from six independent mice (*n* = 6).

### 2.6 Biomechanical recovery of skin strength

Following euthanasia, rectangular section of the healed skin (~2 cm × 1 cm) were excised around the incision area on Days 2 and 7 postclosure to investigate the biomechanical recovery of skin following suturing or laser sealing. In case of suture‐closed incisions, sutures were removed prior to tensile strength measurements in order to obtain strength of the healed skin alone. Excised skin samples were secured in clamps and stretched until failure stretched at a rate of 2 mm/s under constant tension using a TA.XT Plus texture analyzer (Texture Technologies Corp.). UTS was determined from the maximum force of the tissue prior to failure, where the maximum force (*F*) and cross‐sectional area of the tissue sample (*A*, length of skin sample 1 cm and tissue thickness 500 μm) determined the UTS (*σ*, Pa) of healed skin (*σ* = *F*/*A*). The tensile strength of unwounded skin (~2 cm length × 1 cm) was also tested for comparison. All tensile strengths are displayed as mean ± standard error of mean from six independent experiments (*n* = 6).

$$\mathrm{UTS}\;\text{of healed skin}\left(\text{in}\;\mathrm{Pa}\right)=\frac{\text{Maximum force}\;\mathrm{at}\;\text{skin ruptre}\left(\text{in}\;N\right)}{\text{Cross}-\text{sectional area of skin}\left(\text{in}\;m^{2},\text{length}\times \text{thickness}\right)}.$$

Percentage intact skin strength for healed skin were calculated as a difference between UTS for each group either on Day 2 or Day 7 postclosure from unwounded skin strength, with the difference then converted to a percentage.

$$\%\text{intact skin strength}=\begin{array}{l} \frac{\mathrm{UTS}\;\text{of healed skin}\left(\mathrm{on}\;\mathrm{Day}\;2\;\text{or}\;\mathrm{Day}\;7\right)}{\mathrm{UTS}\;\text{of unwounded skin}\left(\mathrm{no}\;\text{incision control}\;\mathrm{on}\;\mathrm{Day}\;2\;\text{or}\;\mathrm{Day}\;7\right)}\times 100. \end{array}$$

### 2.7 US and PAI of LASE‐tissue interface

**FIGURE 1:**
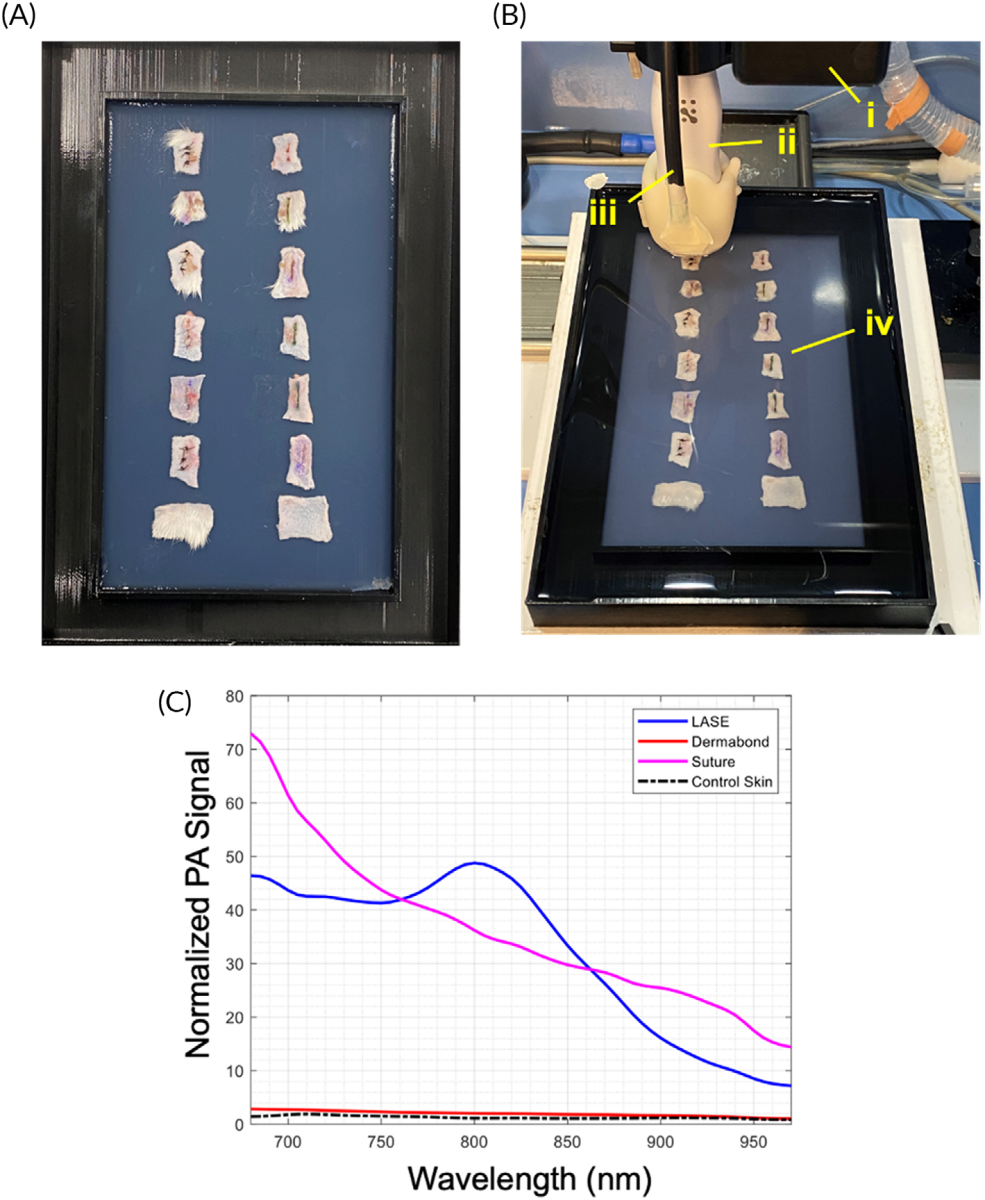
Set up for ultrasound (US) and photoacoustic imaging (PAI) for skin incisions closed with sutures of sealed with LASEs. (a) Prior to imaging, excised skin samples were removed from ice‐cold 1X PBS and placed over a layer of 1.5% agarose low electroendoosmotic (EEO) cooled to room temperature in a 3D printed sample tray. Following this, another layer of agarose solution (~35–45°C) was poured over the skin samples to completely embed the tissues within the agarose layers. The sample tray was then filled with deionized water to form a layer over the agarose layer. (b) (i) Scanning of skin samples were carried out using the Vevo 3100 motor. (ii) The MX550D (50 MHz) linear array transducer and jacket consisting of the fiber bundles (iii) is lowered into the sample tray submerged in water to facilitate opto‐acoustic coupling with a 7 mm standoff from the skin samples (iv). (c) Normalized photoacoustic signal of LASE, suture, and skin sections in the range of 680–960 nm.

Similar to studies on biomechanical recovery of skin, rectangular sections of the healed skin (~2 cm × 1 cm) were excised around the incision area on Days 2 and 7 postclosure, collected in biopsy cassettes, and stored in ice‐cold 1X PBS (10 mM sodium phosphate, 1.8 mM potassium phosphate, 2.7 mM potassium chloride, 137 mM sodium chloride, pH 7.4) prior to US and PAI. All skin specimens were imaged within ~4 h of necropsy and skin collection. Skin specimens were removed from 1X PBS, blotted to remove excess buffer and embedded in a 1.5% agarose gel (Millipore Sigma; #A9539; low EEO) within an in‐house 3D printed tray (Figure 1a). The outer section of the tray was filled with deionized water in order to submerge the agarose layer (Figure 1a). High‐resolution US and PAI were carried out with the MX550D (50 MHz) linear array transducer, fiber bundle and motor setup of the Vevo 3100 + LAZR‐X (VisualSonics) at the University of Arizona, Tucson, AZ. A transducer jacket (VisualSonics; Figure 1bii) was used to combine the transducer (Figure 1bi) and fiber bundle (Figure 1biii), allowing the laser light to be directed to a region 7 mm away from the transducer head. The transducer with jacket was lowered into the water bath to achieve opto‐acoustic coupling for PAI with an optimal standoff required for the LAZR‐X system of approximately 7 mm. The spatial resolution of the imaging system is ~30 microns transverse normal, ~50 μm azimuth, and ~ 300 μm slice thickness. For 3D scanning of skin, the transducer with jacket was moved incrementally at a step size of 150‐μm in the elevational direction across the length of each skin sample (Figure 1biv). For each skin specimen, PA data were obtained at eight wavelengths (40‐nm increments from 680 to 960 nm) for each depth/width cross‐section of the 3D scan, along with a standard pulse echo US image. A center slice for each sample is chosen for full PA spectrum characterization (5‐nm increments from 680 to 960 nm). Furthermore, length/width sample cross‐sections are compiled from the 3D data set in image postprocessing.

Spectral unmixing was performed on the obtained PA signal to discern the tissue constituents. The VevoLAB software (VisualSonics) is utilized for such spectral unmixing, where three wavelength components (680, 800, 960 nm) are used to discern LASE signal from that of the weakly absorbing normal skin. Multi‐wavelength unmixing for ICG content carried out using the Vevo system has been shown previously to produce accurate results for deep tissue imaging in tissue phantoms and murine subjects.^34^, ^35^ Control skin has a relatively weak and flat PA spectrum across the wavelength band, implying that the strong ICG absorption at 800 nm can be used to identify LASE within the samples (Figure 1c), considering that ICG is mixed with silk to form the LASE.

### 2.8 Tissue collection and processing for histology analyses

Following euthanasia, healed tissues were carefully excised, flattened between two foam biopsy sponges in a tissue cassette, and fixed by submersion in 10% neutral‐buffered formalin (#HT501128; Sigma‐Aldrich) for a minimum of 72 h at RT. Tissues were dehydrated through a graded alcohol series and paraffin embedded with Paraplast Plus (#19217; EMS Diasum) by manual processing (Table S1). Individual 5‐μm thick sections were cut with an Accu‐Cut SRM 200 Rotary Microtome (Sakura Finetek USA) and collected on charged glass slides (Hareta, Springside Scientific) in a floating water bath (XH‐1003; IHC World). Slides were dried overnight at 37°C and stored at RT until use.

### 2.9 Hematoxylin and eosin staining

Dried sections on charged glass were deparaffinized and rehydrated thorough xylene and graded alcohols into tap water. Rehydrated sections were submerged in a solution of Hematoxylin (Gill No. 2; #GHS232; Sigma‐Aldrich) for 3 min, differentiated by 6–12 quick dips in acid alcohol (0.3% hydrochloric acid in 70% ethanol), and blued in a solution of ammonium water (0.2% ammonium hydroxide in distilled water) for 30 s. Slides were further submerged in a solution of 0.5% Eosin Y (#318906; Sigma‐Aldrich) in distilled water (acidified with 0.2% glacial acetic acid vol/vol) for 4 min followed by dehydration through 90% and absolute ethanol, further dehydrated in two changes of 100% xylene, dried and mounted in CytoSeal XYL (Richard‐Allan/Thermo Fisher Scientific). Samples were imaged on an Olympus BX43 upright microscope equipped with an Olympus DP74 CMOS camera operated by cellSens Standard software (Olympus Corporation).

### 2.10 Picrosirius red staining

Dried sections on charged glass were deparaffinized and rehydrated thorough xylene and graded alcohols into tap water. Rehydrated sections were submerged in a 0.1% solution of Picrosirius Red composed of Direct Red 80 (#365548; Sigma‐Aldrich) in a saturated aqueous solution of picric acid (#P6744; Sigma‐Aldrich) for 1 h at RT to achieve stain saturation. Slides were washed twice in acidified water (0.5% glacial acetic acid in distilled water) for 2 min each. Slides were dehydrated through an abbreviated 90% and absolute ethanol series, further dehydrated in xylene and mounted in CytoSeal XYL (Richard‐Allan/Thermo Fisher Scientific). Brightfield images were collected on an Olympus BX43 upright microscope equipped with an Olympus DP74 CMOS camera operated by cellSens Standard software (Olympus Corporation).

### 2.11 Immunohistochemistry

Captured sections were rehydrated and overnight epitope retrieval was performed in sodium citrate buffer at 60°C. Tissue sections were blocked with 5% BSA in TBS containing 0.2% Tween‐20 (TBST) at RT for 1 h and incubated overnight with primary antibodies for iNOS (Abcam; ab15323; rabbit polyclonal; 1:50), Arginase‐1 (Cell Signaling Technologies; #93668; rabbit monoclonal; 1:200), or Ly6G (Invitrogen; #14‐5931‐82; rat monoclonal; 1:100). Secondary antibodies were probed for 2 h at RT using HRP‐(rabbit) or AP‐(rat) conjugates (Jackson Immunolabs). Tissues were developed with ImmPACT DAB (HRP) or Vector Red (AP) substrate (Vector Labs). Arginase‐1 and Ly6G sections were counterstained with hematoxylin while iNOS sections were left without counterstain. Tissues were dehydrated through alcohol and xylene and mounted with Cytoseal XYL. Images were collected on an Olympus BX43 upright microscope equipped with an Olympus DP74 CMOS camera operated by cellSens Standard software. Images were quantified in ImageJ/FIJI.

### 2.12 Image analyses

Morphometric features of healing were assessed in ImageJ/FIJI. Images were calibrated according to magnification. Epidermal gap was measured as the linear distance between the two epidermal faces of the wound edges and identified by canonical appearance of the epidermis via hematoxylin and eosin (H&E) staining on Days 2 and 7 postinjury. Histological scar area was measured as the area weakly stained by Picrosirius Red and bounded by the basement of the epidermis and above the hypodermis, and the edges of the mature collagen in periwound tissue strongly stained by Picrosirius red on Day 7 postinjury.^36^ Dermal gap was measured as the linear distance between intact areas of dermis, indicated by bundled collagen fibers.

### 2.13 In vivo live mouse US of sealed wounds

Mice (2‐day post‐incision) were anesthetized using ketamine/xylazine cocktail. Once under surgical anesthesia (confirmed by toe pinch), mice were placed on a heated mat and imaged by US with a GE Logiq *e* Nextgen ultrasound system equipped with a 10–22 MHz transducer (Figure S1). Mice were euthanized postimaging and tissues were fixed and processed as described below. US data were transferred into ImageJ/FIJI and calibrated according to the length of the transducer's dimensions (19.3 mm field width). To enhance better feature visualization, the US images were normalized by using an ImageJ native *Bandpass Filter* function, and epidermal/dermal gap was quantified by linear measurement. H&E‐stained images for matched mice were evaluated and epidermal/dermal gap was quantified by linear measurement. Correlation between B‐mode US images acquired in vivo and H&E histology for Day 2 incisions was evaluated by simple linear regression with 95% confidence interval in GraphPad Prism.

### 2.14 Statistical analyses

Data from absorbance, TEWL, and skin UTS are presented as mean ± standard error of the mean. Differences between groups were assessed using two‐way analysis of variance followed by Fisher's LSD test using GraphPad Prism version 9.2.0 (GraphPad). A *p* < 0.05 was considered statistically significant.

## 3 RESULTS AND DISCUSSION

Laser sealing is an attractive approach for the sutureless approximation of tissues, including skin, and possesses several potential advantages including fast operation times, low scarring, and faster recovery of tissue function. However, the temporal dependence of the efficacy and quality this approach has not been investigated thoroughly. We, therefore, carried out detailed studies to investigate the efficacy of laser sealing in live mice in a temporal manner and compared findings with those seen with sutures. In addition to biomechanical recovery with UTS, functional recovery of barrier function of skin (using TEWL measurements), US and photoacoustic visualization and histology studies were used to develop a more comprehensive investigation into the quality and efficacy of tissue repair following laser sealing.

### 3.1 Generation and characterization of LASE films

**FIGURE 2:**
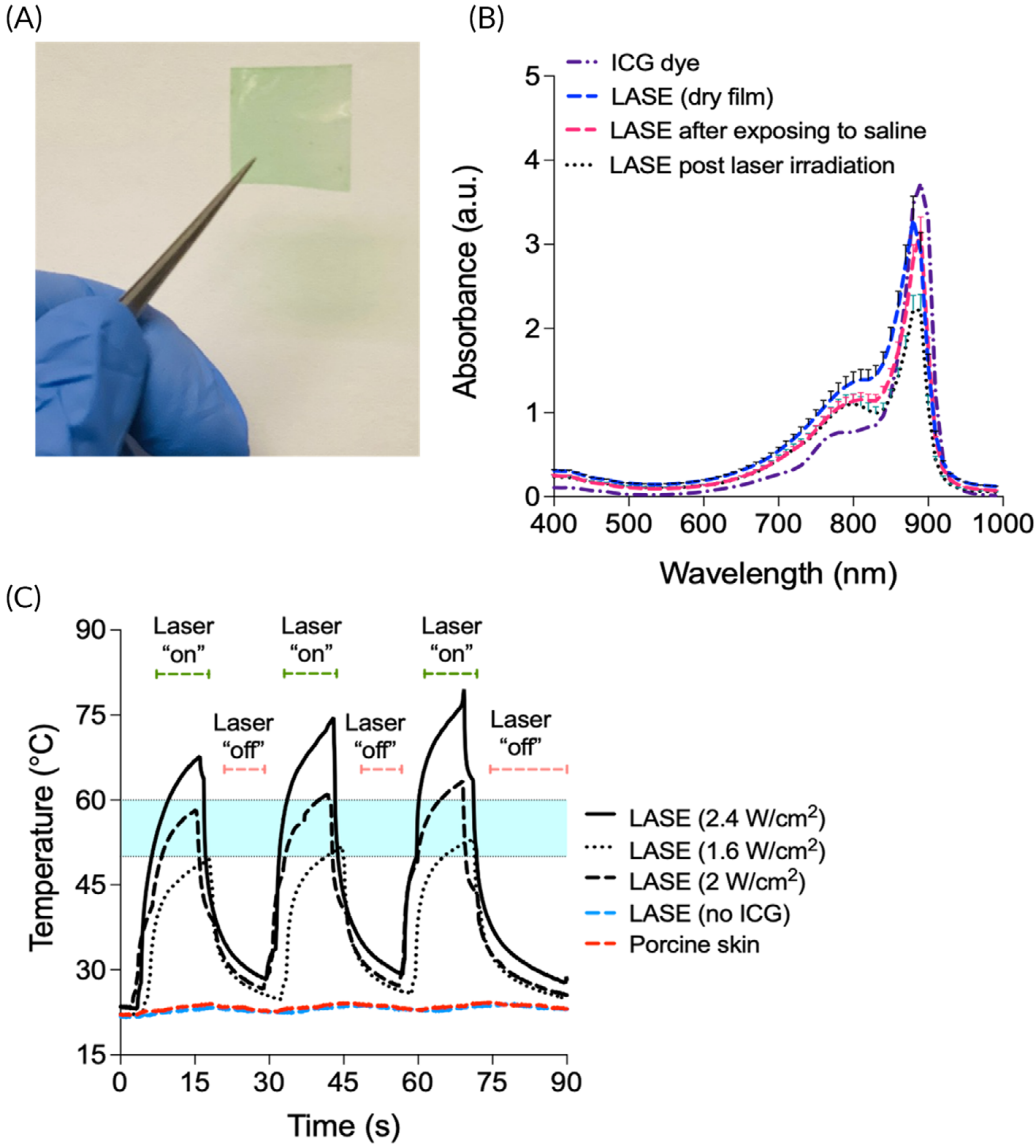
Silk‐ICG laser‐activated sealants (LASEs). (a) Representative image of a 2 cm × 2 cm LASE film fabricated from silkworm silk fibroin and indocyanine green (ICG) dye; the green color of the LASE is because of the ICG dye. (b) Absorbance spectra of ICG dye alone (dashed and dotted purple line), LASE film—as it is fabricated (dashed blue line), LASE in a viscous paste form after addition of saline, which was used to mimic a moist environment in wound beds (dashed red line), and post‐laser irradiation in the paste form (dotted black line). Data shown are mean ± standard error of the mean of *n* = 4 independent LASE films. (c) Photothermal response of LASE on ex vivo porcine skin irradiated using a continuous wave NIR laser tuned to 808 nm at varying laser power density from 1.6 to 2.4 W/cm^2^ in a 15 s “on” and 15 s “off” cycle (3 cycles total). Photothermal responses of silk films (with no ICG dye added, red dashed line) and porcine skin (blue dashed line) following irradiation with the laser at 2.4 W/cm^2^ are also shown. The region shaded in light blue color (temperature range from ~50°C to ~60°C) indicates the optimal temperature window for laser tissue sealing. Each photothermal response curve is a mean of *n* = 3 independent experiments.

Silk fibroin (“silk”)‐ICG LASE films (Figure 2a) were prepared using solvent evaporation methods as described in our previous reports.^15^ Briefly, aqueous solutions of silk fibroin (6 wt% or 60 mg/ml) with 0.1 mM ICG were cast and dried overnight at RT resulting in the generation of LASE films following solvent evaporation. Light absorption analyses indicated that the LASE films displayed a characteristic absorbance similar to that of ICG dye (Figure 2b). Upon addition of saline solution, LASE films dissolve to form an adhesive viscous paste, which is suitable for sealing incisional wounds. The viscous paste maintains absorbance properties in the NIR window as seen in case of dry LASE films. This viscous adhesive paste was irradiated using a hand‐held 808 nm continuous wave laser under conditions (power output ~100 mW, power density ~3.2 W/cm^2^, 1 min, LASE surface temperature ~50–60°C) similar to those used for in vivo skin sealing. No significant shifts in absorbance profiles of these irradiated viscous LASE pastes were seen (Figure 2b). The retention of absorbance properties by laser‐irradiated LASEs warrants the use of optical visualization methods for probing LASE following tissue sealing in subsequent applications.

We also investigated the photothermal response of LASE films irradiated with a NIR (808 nm), continuous wave hand‐held laser turned on for 15 s (“on” cycle) and off for 15 s (“off” cycle) for a total of 3 cycles. For photothermal response studies, a LASE section was applied to an ex vivo porcine skin where the LASE section turned into a viscous paste upon contact with the skin and the surface temperature was recorded using an IR camera. Upon irradiation with a laser, a rapid increase in surface temperature of the LASE‐tissue interface was observed due to efficient photothermal conversion of the embedded ICG dye in the LASE matrix. This photothermal response was reproducible over 3 cycles and varied using different laser power densities (1.6–2.4 W/cm^2^) (Figure 2c). Irradiation of porcine skin alone and LASE without ICG even at the highest laser power density tested (2.4 W/cm^2^) did not result in any increase in temperature (red and blue dotted line, respectively, Figure 2c). Surface temperatures in the range of 50–60°C (shown using blue shaded region) optimal for tissue sealing were achieved by modulating the laser power density (Figure 2c).

### 3.2 In vivo sealing of skin incisions: Barrier function recovery and healed skin strength

**FIGURE 3:**
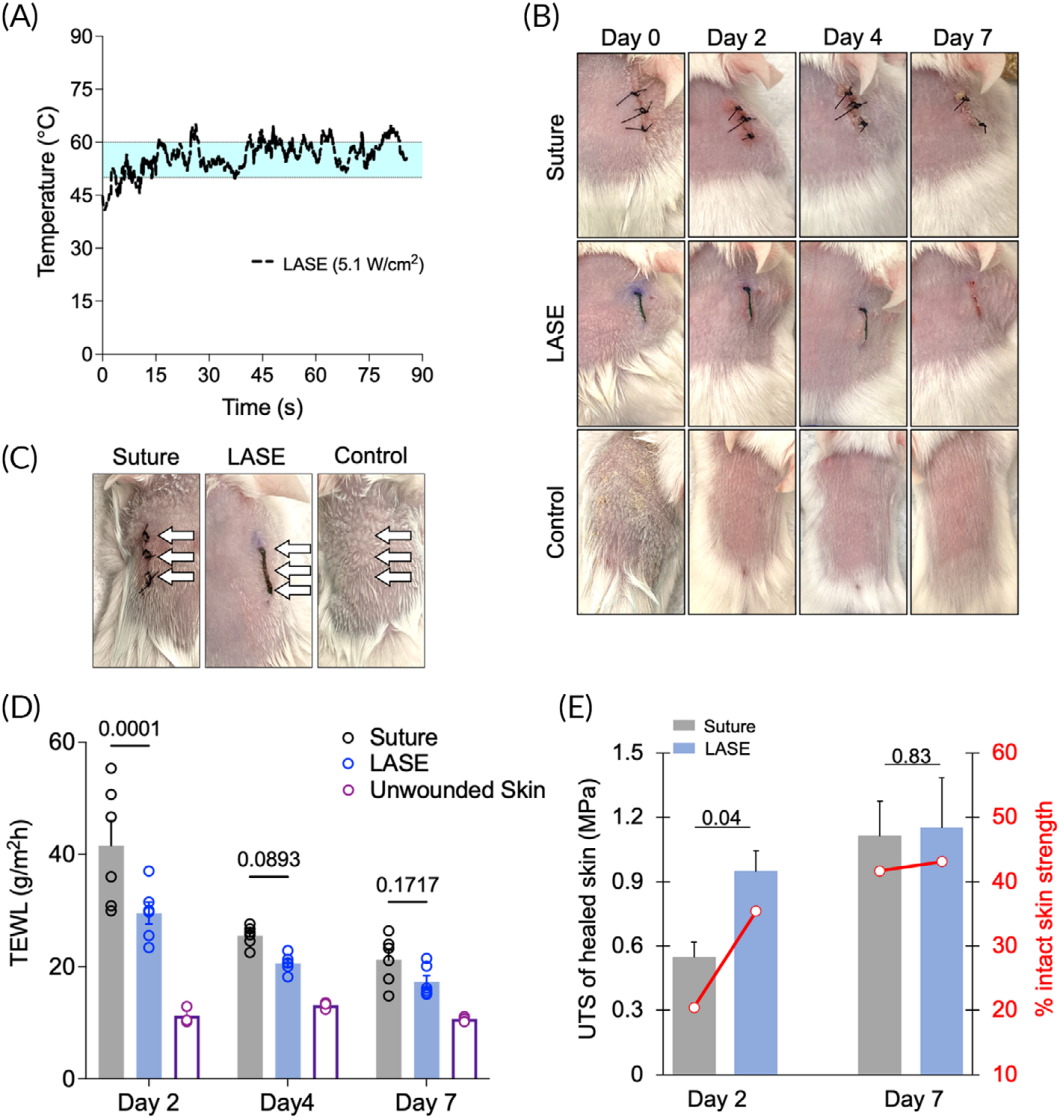
Functional and biomechanical recovery of skin following suture closure and LASE sealing in Balb/c mice. (a) Photothermal response of LASE‐skin interface during in vivo sealing irradiated using a continuous wave NIR laser tuned to 808 nm at a laser power density of ~5.1 W/cm^2^. The region shaded in light blue color (temperature range from ~50°C to ~60°C) indicates the optimal temperature window for laser tissue sealing. The photothermal response curve shows data that are a mean of *n* = 3 independent experiments. (b) Representative images of 1‐cm long skin incisions closed with four, simple interrupted 4‐0 nylon sutures or LASE on Days 0 (immediately after closure), 2, 4, and 7 postwounding; control is unwounded skin surgically prepared similarly to incised skin. (c) Representative image showing three approximate locations at which TEWL measurements were carried out (white arrows) for each type of closure method. (d) Transepidermal water loss (TEWL) of healed skin and unwounded control skin on Days 2, 4, and 7 postwounding. TEWL value (in g/m^2^ h) for each incision type is the average TEWL measurement from three nonoverlapping spots over the incision line shown in b. Data shown are mean ± standard error of the mean of *n* = 6 mice. (e) Ultimate tensile strength (UTS) and recovery, that is, %UTS of intact skin strength (secondary axis shown in red) of healed skin on Days 2 and 7 postwounding for suture‐closed and LASE‐sealed incisions. Data shown are mean ± standard error of the mean of *n* = 6 mice. Statistical significance was determined using two‐way ANOVA followed by Fisher's LSD test and individual *p* values are shown; *p* < 0.05 are considered statistically significant.

Full‐thickness incisional dorsal skin wounds in BALB/c mice, 1‐cm in length, were sealed using LASE or approximated with 4‐0 Nylon sutures. In the case of laser‐sealed incisions, the LASE‐tissue interface (incision line) was irradiated for 1 min at a power density of ~5.1 W/cm^2^ (~160 mW power output, ~2 mm laser beam diameter), corresponding to temperatures in the range of 50–60°C at the skin−LASE surface (Figure 3a). Mice were allowed to recover and representative images of incisions on Days 0 (immediately after surgery), 2, 4, and 7 are shown in Figure 3b. Mice without incisions were surgically prepped, recovered in a similar manner, and were used as controls in subsequent studies. Following wounding and closure of skin incisions, barrier function recovery of the healing skin was determined in a noninvasive manner using measurements for TEWL. TEWL is a marker of skin permeability which measures water loss thorough the stratum corneum layer and is one of the standard methods to evaluate barrier function of skin. Any damage or trauma to the skin barrier leads to an increase in TEWL levels compared to intact or unwounded skin.^32^ TEWL levels of the 1‐cm long incisions closed with LASE or sutures were determined on Days 2, 4, and 7 postsurgery from three nonoverlapping regions (shown by white arrows) over the incision line (Figure 3c). The skin in the incisional region closed with LASE or sutures demonstrated gradual decrease in TEWL values. At Day 2 postwounding, the average TEWL value for incisions closed with LASE (29.5 ± 1.9 g/m^2^ h) was significantly lower than sutured incisions (41.6 ± 4.4 g/m^2^ h; *p* = 0.0001). On Days 4 and 7 postwounding, TEWL values of LASE‐sealed incisions were lower compared to those with sutures (Day 4: 20.6 ± 0.6 vs. 25.5 ± 0.7 g/m^2^ h; *p* = 0.0893 and Day 7: 17.3 ± 1.1 vs. 21.2 ± 1.7 g/m^2^ h; *p* = 0.1717), but the differences were not statistically significant (i.e., *p* values were not <0.05). Average TEWL values of unwounded skin on Day 2, 4, and 7 postwounding were considered as baseline on those corresponding days (Figure 3d).

In the above TEWL studies, Days 2, 4, and 7 postinjury were chosen in order to investigate early, mid, and later stages of wound repair following closure by primary intention as in case of incisional wounds. The time point of Day 2 postinjury is a good temporal representative of the early inflammatory phase, which helps with clearance of tissue debris from injury and kickstarts processes that prepare the wound for subsequent stages of repair, that is, proliferation and remodeling. Day 7 postinjury was chosen as a likely representative of the later remodeling phases, and Day 4 likely captures proliferation and/or potentially the transition from the proliferation stage to the later remodeling stage. During proliferation and remodeling stages, deposition of collagen matrix, angiogenesis, and maturation of granulation tissue are key events, and newly deposited matrix leads to an increased tensile strength of healing wounds. This phase can vary in length based on the wound site, tissue type, and type of injury. Mouse skin heals by contraction, which is different from skin healing by re‐epithelialization in humans. Contraction‐facilitated healing in mice shows faster kinetics of closure (e.g., over a 4–7‐day period) compared to wound healing dynamics seen in humans. To that end, interrogation at an earlier time point, that is, Day 2, can lead to meaningful insights into the efficacy of different wound approximation devices including sutures and LASEs.

Mice were euthanized on Days 2 or 7 postwounding to evaluate biomechanical recovery of the healing skin both at an early and late time point. At the earlier healing timepoint (Day 2 postwounding), LASE‐sealed incisions had higher UTS (0.87 ± 0.13 MPa) compared to sutured incisions (0.47 ± 0.08 MPa; *p* = 0.0464). The UTS of unwounded (no incision control) skin of BALB/c mice of the same age range was 2.67 ± 0.17 MPa and was used to compare the efficacy of healing using sutures and LASE. Incisions closed with sutures and LASE resulted in a UTS recovery of approximately 20.4 ± 2.6% and 35.4 ± 3.6%, respectively, relative to that of intact skin on Day 2 postclosure (secondary axis in Figure 3e). Our results indicate improved efficacy in recovering the skin tensile strength at an earlier timepoint with LASE, which is consistent with our previous observations with silk‐GNR gold nanorod sealants for incisional skin repair.^14^ At the latest healing time point (Day 7 postwounding), UTS of LASE‐ and suture‐closed skin increased to 1.15 ± 0.23 and 1.11 ± 0.16 MPa, respectively (not significant; *p* = 0.8720). At this time point, suture and LASE closures resulted in a recovery of approximately 41.6 ± 4.9% and 43.1 ± 7.1% in UTS, respectively, relative to that of intact skin (Figure 3e; secondary axis). This is likely because of the contractile forces in mice skin that aid healing at later durations postsurgery.

Effective functional and biomechanical recovery of skin at early times following injury or surgery is critical particularly considering that different pathologies influence the rate of wound healing. For example, diabetic humans and mice demonstrate delayed wound healing. To that end, our approach of following the efficacy of incisional wound healing using TEWL measurements with time is well‐suited to address temporal progress of healing including in different pathologies that influence tissue repair. For slower healing wounds, faster closure and effective tissue repair are imperative in order to prevent infections and further complications. To that end, the LASE approach, which shows better barrier function (TEWL) and biomechanical (UTS) recovery at earlier time points (Day 2), has the potential to also engender better outcomes in hosts with slower healing wounds.

### 3.3 US and PAI

**FIGURE 4:**
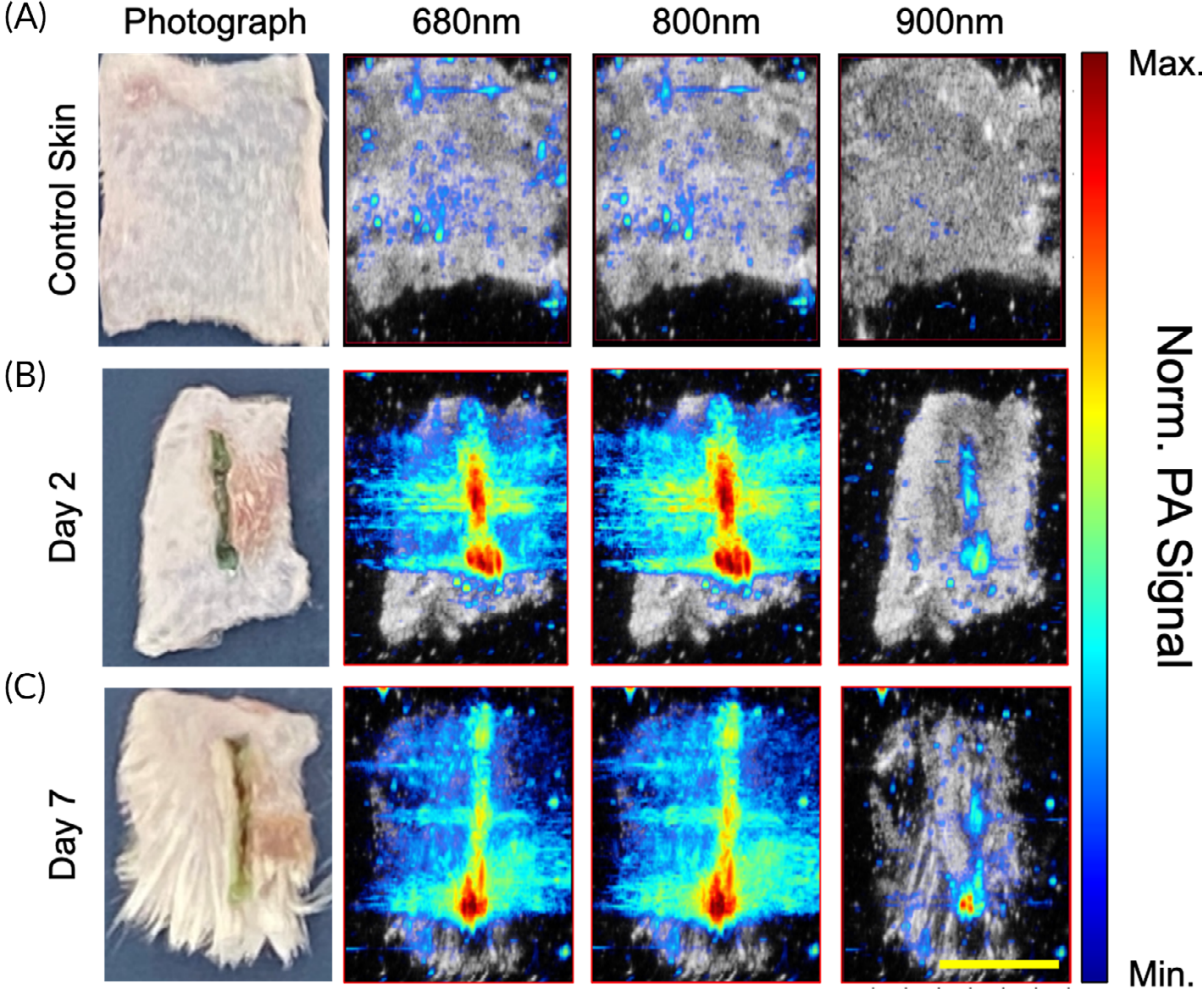
Normalized sample PA signal. Computed transverse slices of B‐mode scans co‐registered with normalized PA signal at 680, 800, and 960 nm for (a) control skin without any incision surgically prepped similarly to skin samples with incisions (b) skin incisions sealed using LASE at Day 2 postclosure and sealing (c) skin incisions sealed using LASE at Day 7 postclosure and sealing. Co‐registered B‐Mode and PA images are obtained by selecting a slice from the 3D scan data set that corresponds with approximately 500 μm subsurface depth (scale bar in yellow = 5 mm).

**FIGURE 5:**
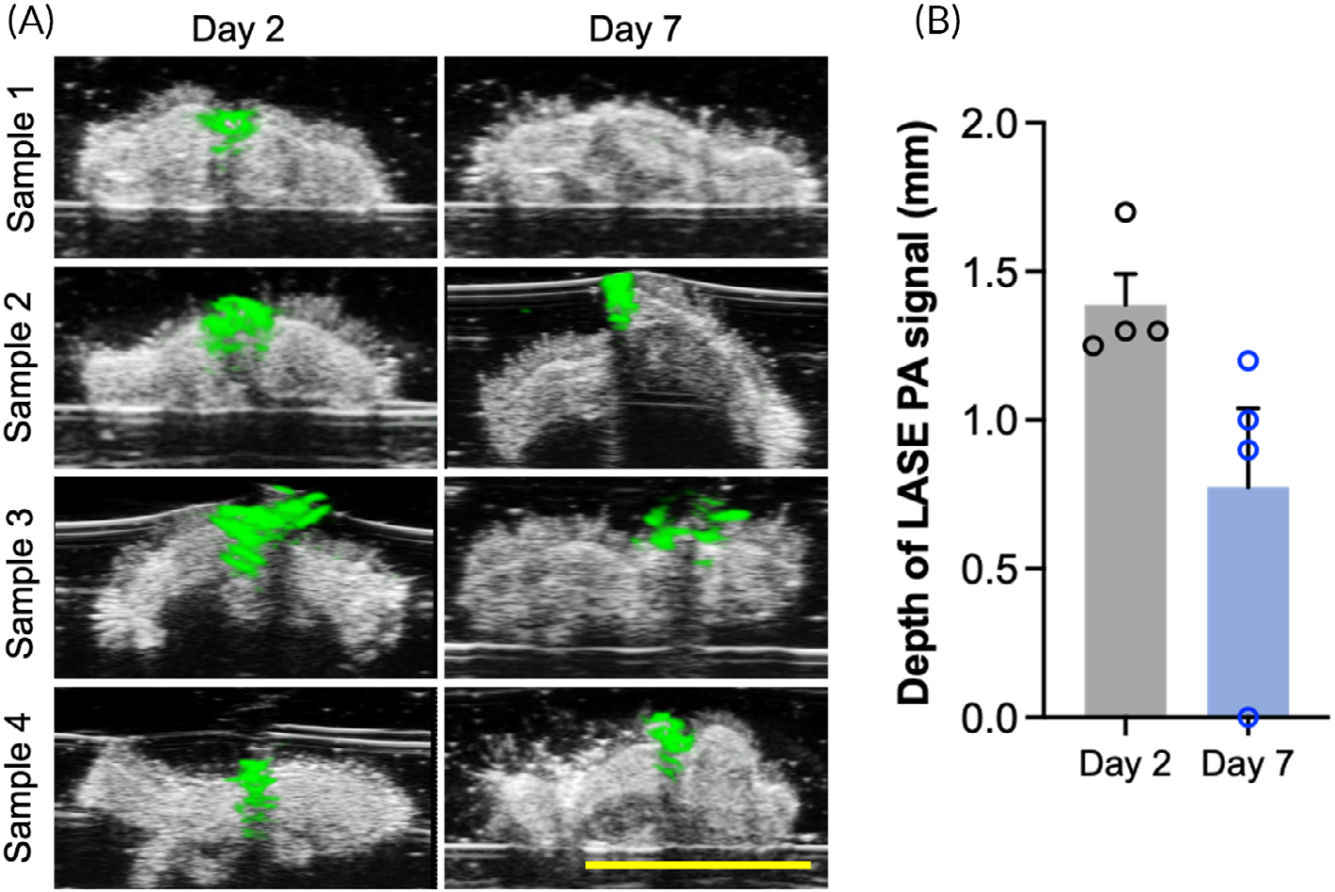
Depth profile of LASE in wound bed. (a) Cross‐section of B‐mode scans superimposed with the normalized PA signal from LASE at 800 nm shown in green for skin samples at Day 2 postclosure and Day 7 postclosure. (b) The depth profiles of photoacoustic signal from LASE in the wound are represented as mean ± standard error of mean of *n* = 4 LASE‐sealed skin samples at each timepoint (scale bar in yellow = 5 mm).

For PAI, a full spectrum scan between wavelengths of 680–980 nm, with a 5‐nm increment, was carried out for a representative center slice of every skin sample. LASE PA signal shows a maximum at 800 nm, which is expected given the absorption of ICG. Control skin displays weak PA signal across all wavelengths, while the black‐colored suture produces strong broadband signal. For PAI and spectral unmixing, signal data must be acquired at minimum of three wavelengths to distinguish three separate constituents (i.e., skin, suture, LASE). Observing the PA signal spectrum of each constituent, wavelengths of 680, 800, and 960 nm are chosen for the spectral unmixing. A 3D scan is then carried out for each sample with data being acquired at the three unmixing wavelengths (Figure 4a–c). It was qualitatively observed that there was a considerable drop in normalized PA signal at 800 nm on Day 7 postclosure compared to Day 2 postclosure (Figure 4b,c). This is further observed as reduced PA signal from LASE identified by the spectral unmixing technique on Days 2 and 7 postclosure. The maximum depth of penetration of LASE signal in the wound bed from four independent LASE‐sealed skin incisions was calculated from the normalized PA signal overlaid on individual US images at both the timepoints (Figure 5a). The average depth of the LASE signal in the wound bed was 1.4 ± 0.2 and 0.6 ± 0.45 mm on Day 2 and Day 7 postclosure samples, respectively (Figure 5b). The PA signal depth can be indicative of persistence of LASE in the wound bed as the healing of the incisional wound progresses over time.

### 3.4 Histological evaluation of LASE‐sealed and suture‐closed skin sections

**FIGURE 6:**
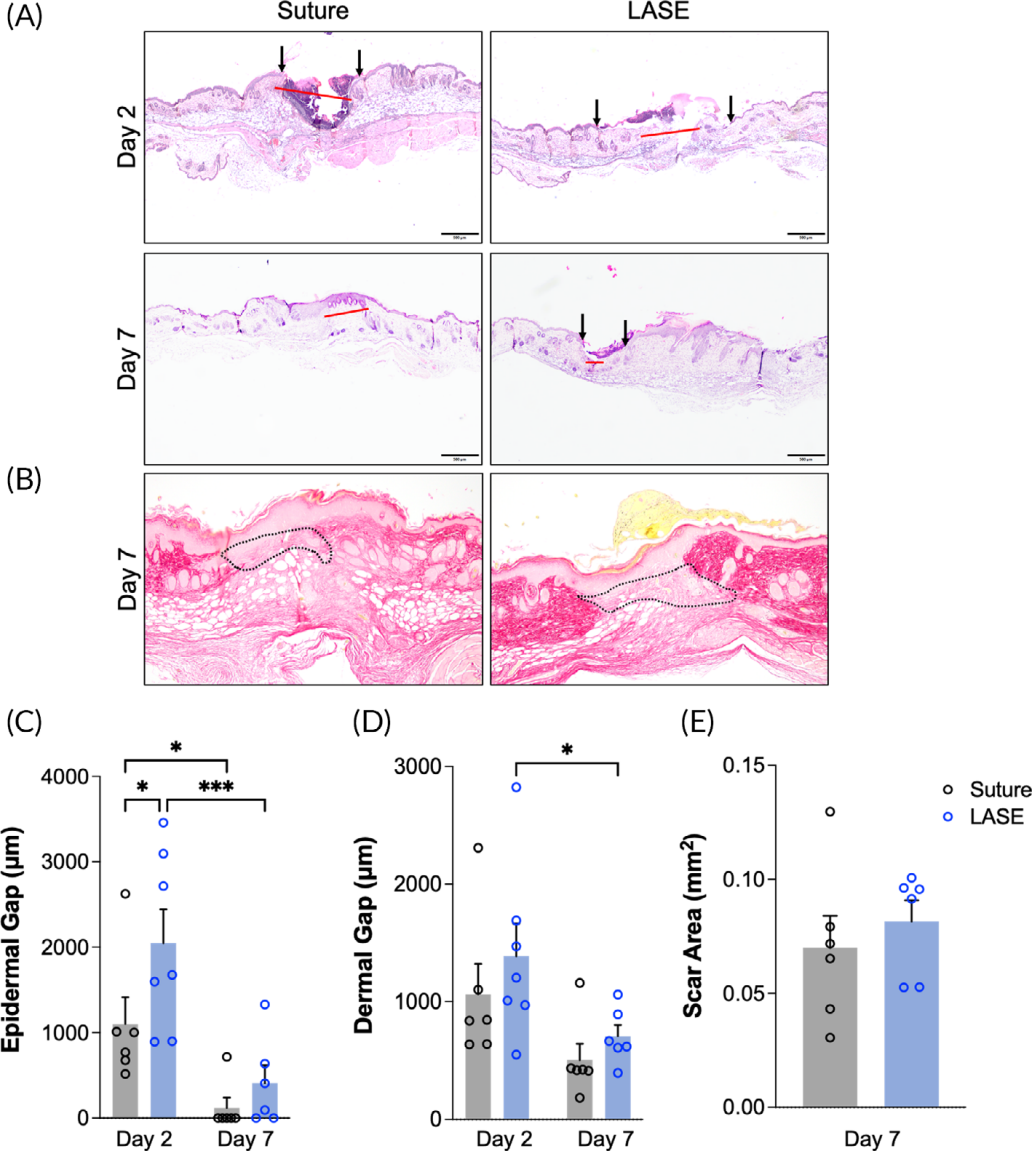
Histological evaluation of skin sections during the course of healing following closure with sutures or sealing with LASE. (a) Representative hematoxylin and eosin (H&E) stained micrographs of the wound sections (×4 magnification) showing the epidermal gap (black arrows) and dermal gap (red line) on Days 2 and 7 postclosure (scale bar = 200 μm). (b) Representative picrosirius red stained micrographs of the wound sections (×10 magnification) showing the scar area in the granulation tissue (black dotted line area) at Day 7 post closure. (c and d) Quantification of epidermal gap and dermal gap in skin sections (in μm) closed with suture or LASE on Days 2 and 7 postclosure. (e) Quantification of histological scar area (in mm^2^) in skin sections closed with suture and LASE on Day 7 postclosure. Data shown are mean ± standard error of mean of *n* = 6 mice per group. Statistical significance was determined using two‐way ANOVA (for epidermal gap quantification) and one‐way ANOVA (for scar area quantification) with Fisher's LSD post hoc analysis. *p < 0.05 is considered significant.

During wound healing, re‐epithelialization is a crucial step for restoring barrier function and preventing exposure to pathogens that cause surgical site infections.^37^ We visualized the cellular and tissue processes that lead to skin healing using a histological analysis (Figure 6a,b). At Day 2 postclosure, suture‐closed skin incisions had a lower epidermal gap compared to LASE‐sealed incisions (Figure 6a,c). The increased epidermal gap seen in LASE‐sealed incisions may be attributed to heat ablation of keratinocytes in the immediate periphery of the LASE.^13^ However, we observed a continuity of closure in the LASE‐sealed wounds, despite the difference in epidermal gap, due to the occupancy of the gap by the LASE material itself, analogous to an eschar. By Day 7 postclosure, no significant difference in epidermal gap was observed between the two groups. Dermal gap (the distance between the collagen fronts of the dermis at the wound edge) was not different between suture of LASE‐sealed wounds at Day 2 (*p* = 0.2870) or Day 7 (*p* = 0.5216), although as expected there was a reduction of dermal gap within the treatment groups between Days 2 and 7 for suture (*p* = 0.0867) and LASE (*p* = 0.0331) (Figure 6a,d). We also evaluated the histological scar area, observed through picrosirius staining, to determine if there is a difference in initial scarring during the healing period in incisions closed with suture or LASE (Figure 6b,e).^38^ Histological scar areas of 0.07 ± 0.01 and 0.08 ± 0.01 mm^2^ (*p* > 0.05) were seen in case of suture‐closed and LASE‐sealed incisions, respectively, at Day 7 postclosure, indicating that both resulted in similar levels of scarring based on these analyses (Figure 6e). Nascent collagen deposition was not appreciable on Day 2 postclosure; thus, no scar area was yet present (data not shown). This is expected considering the longer timeline necessary for development of scar‐like formation. Taken together, these results indicate that LASE‐sealed incisions exhibit rapid and robust sealing with minimal effect on scarring or tissue integrity, while also providing an improvement in early barrier function and tissue strength. While both sutures and LASE‐sealed incisions exhibited some degree of epidermal and dermal gap, sutures are an interrupted sealing method (i.e., sutures have empty space between individual placements) and tissue puckers and is open between each knot. LASE, on the other hand, provide a continuous seal across the length and width of the incision, bridging the tissue space and providing a more complete protection from the environment, in a manner similar to a natural eschar, but on‐demand and with high strength. This, in part, contributes to the higher biomechanical and functional recovery seen with LASE sealing.

**FIGURE 7:**
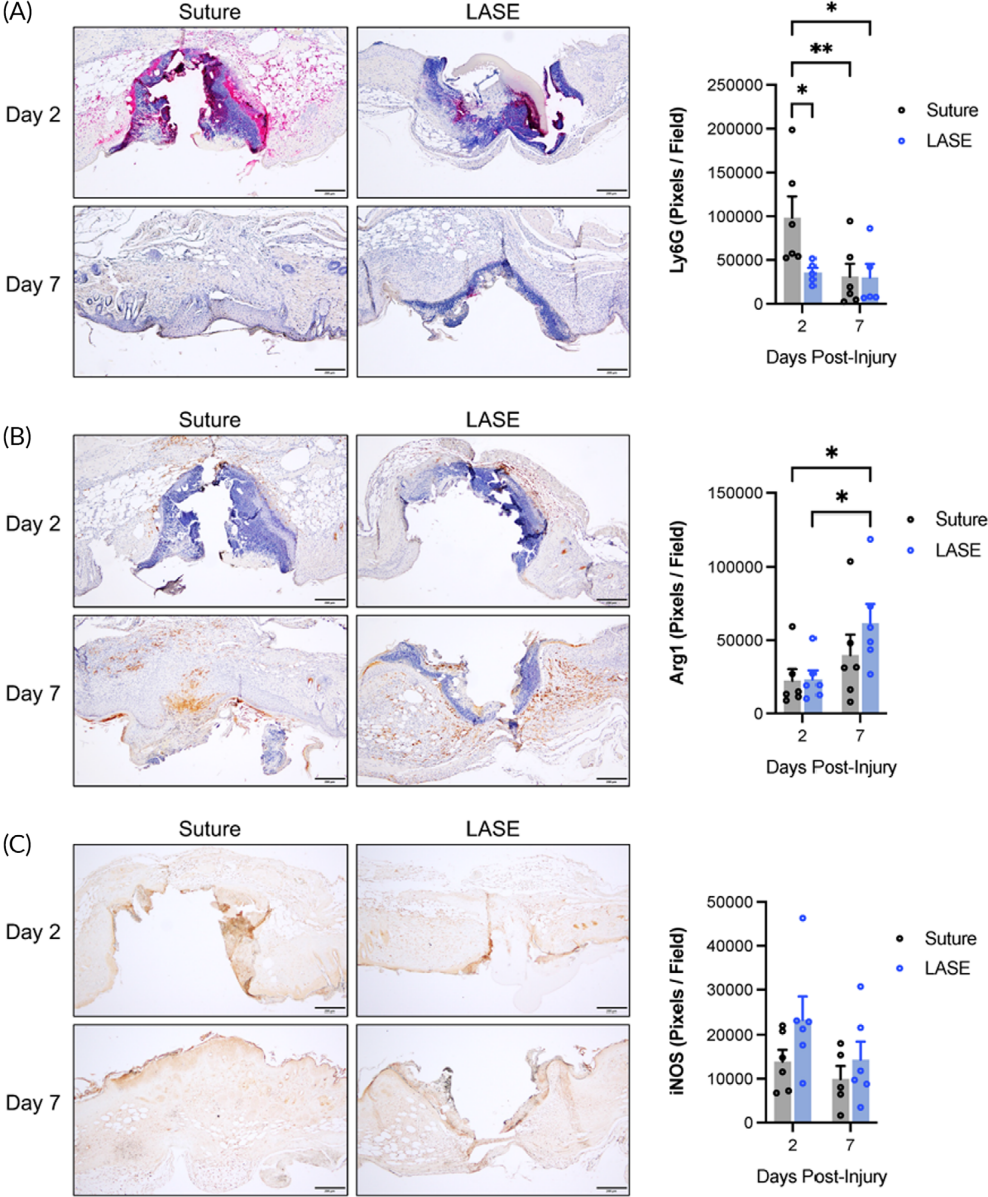
Immunohistochemical evaluation of incised skin during the course of healing. (a) Representative micrographs and quantification of wound sections (×4 magnification) stained for Ly6G (pink chromogen) with hematoxylin counterstain at Days 2 and 7 postclosure (scale bar = 200 μm). (b) Representative micrographs and quantification of wound sections (×4 magnification) stained for Arginase‐1 (brown chromogen) with hematoxylin counterstain at Days 2 and 7 postclosure (scale bar = 200 μm). (c) Representative micrographs and quantification of wound sections (×4 magnification) stained for iNOS (brown chromogen) without nuclear counterstain at Days 2 and 7 post closure (scale bar = 200 μm). Data shown are mean ± standard error of mean of *n* = 5–7 mice per group. Statistical significance was determined using two‐way ANOVA (for epidermal gap quantification) and one‐way ANOVA (for scar area quantification) with Fisher's LSD post hoc analysis. Significance indicated as *p < 0.05; **p < 0.01.

Immunohistochemical analysis of tissue sections indicated that wounds treated with LASE had a significant reduction in Ly6G‐positive infiltrating neutrophils at 2‐day postclosure (*p* = 0.0192) (Figure 7a). While there was a nonsignificant trend toward increased iNOS‐positive proinflammatory macrophages at 2‐day postclosure (*p* = 0.0940) and Arginase‐1‐positive proresolution macrophages at 7‐day postclosure (*p* = 0.1672) (Figure 7b,c), we observed an enhancement of Arginase‐1 response (proresolution macrophages) at 7‐day postclosure versus 2 days with LASE (*p* = 0.0207) which did not reach significance for sutures (*p* = 0.2693). These data indicate that sealing wounds with LASE induces an augmentation of immune cell behavior at early and late stages of wounding, with an enhancing effect on the arginase‐1‐positive prohealing macrophage response and a distinct effect on the dynamics of infiltrative neutrophils. The role of neutrophils in healing wounds is evolving, with recent evidence suggesting both positive and negative roles in regulating the healing process.^39^ While neutrophils play an early role in protecting against infection, they are also drivers of early signals to stimulate repair. Neutropenia is associated with slower healing and deficiency of several signals involved in neutrophil function can result in impaired healing.^40^, ^41^, ^42^, ^43^ Conversely, persistence of neutrophils within a wound can delay healing and an overabundance of neutrophil‐derived PAMPs, such as neutrophil extracellular traps (NETs), can lead to chronic wounds and have become therapeutic targets to improve wound healing.^44^, ^45^, ^46^, ^47^ Here, we show that neutrophils are present in both sutured and LASE‐sealed wounds at Day 2, but in much lower abundance in LASE‐sealed wounds, with levels equalizing by Day 7. Most studies investigating the role of neutrophils in wound healing utilize excisional wounding models, which proceeds by secondary intention healing (granulation). Incisional wounding, performed here, proceeds by primary intention healing and may utilize different biological mechanisms. Our finding that reduced neutrophils in LASE‐sealed incisional wounds compared to sutured incisional wounds is in agreement with a recent study by Heuer et al.,^50^ which showed that mouse laparotomy wounds (primary intention healing) treated with DNase I (to deplete NETs) or with PAD4 knockout (to genetically inhibit NET formation) exhibited significant improvements in healing quality. Thus, a controlled or tuned down neutrophil response—as likely induced by LASE sealing—may positively affect incisional (primary intention) wound healing.

### 3.5 US evaluation of in vivo sealed incisions in live mice

**FIGURE 8:**
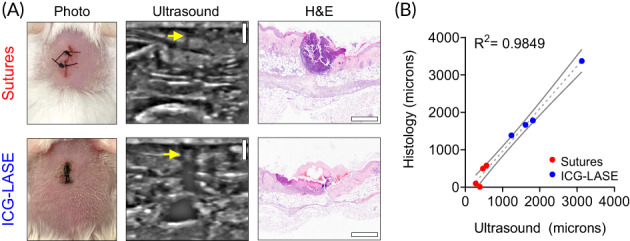
Live animal ultrasound evaluation of suture‐ and LASE‐sealed wounds. (a) Representative photographs, ultrasound imaging data, and H&E images for suture‐ and LASE‐sealed linear incisions imaged by ultrasound in the live animal at Day 2 postclosure. (b) Linear correlation with 95% confidence intervals for the epidermal/dermal gap of wounds sealed by sutures (red) or ICG LASE materials (blue) measured by ultrasound (*x*‐axis) or histology (*y*‐axis). *N* = 4 per group.

We sought to evaluate the fidelity of US to interrogate LASE‐ and suture‐sealed incisions in live mice using a portable, clinical US system; the portable nature and clinical application of this system was considered useful for potential translational applications. Live, anesthetized mice were evaluated with a linear probe transducer in B‐mode operating at 22 MHz. Manually collected US images were compared to matched H&E‐stained sections from the same mice and the linear dimension of the wound width was compared (Figure 8). We found a high degree of correlation between US and histopathology measurements (linear regression *y* = 1.140*x* − 182.5; *R* ^2^ = 0.985, *N* = 4 each group). Thus, in vivo US, using a clinically relevant system, provides an accurate representation of wound properties in mice with incisions sealed by both sutures and LASE.

## 4 CONCLUSION

Tissue adhesives are an alternative and effective method of skin closure following surgical incisions or traumatic lacerations. Here, we comprehensively evaluated the efficacy and quality of silk fibroin‐ICG based LASE for rapid sealing of skin incisional wounds in mice compared to conventional suturing using a temporal study of functional, biomechanical, and histological evaluation in addition to US and PAI. We evaluated healing outcomes at different timepoints in the repair process and our results show LASE‐sealed incisions had earlier recovery of skin barrier function compared to suture‐closed incisions as indicated by lower TEWL rate. At the same timepoint, significant increase in biomechanical recovery of skin was observed in case of LASE‐sealed incisions compared to suture‐closed incisions. Higher biomechanical and functional recovery of skin can prevent dehiscence of wounds early in the healing period and also protect against surgical site infections. US and PAI of skin incisions closed with sutures and sealed with LASE demonstrated that these structures can be identified by their unique optical absorption properties and help quantify and track their presence within a sample volume at least several days postsurgery. The noninvasive dual modality platform can potentially be applied in vivo to track these changes at the skin interface over time. Histological analyses of skin at the end of the healing period in our study (Day 7 postclosure) showed no difference in epidermal gap and scar area compared to suture‐closed incisions which can be indicative of no excessive scarring or fibrosis in using LASE as a skin closure method. However, it is important to note that mouse skin heals by contraction, which poses significant limitations in using mouse models as indicators of scarring. Further studies in relevant animal models (e.g., porcine models) will be key to further compare scarring caused by sutures and LASEs. Evaluation of translational potential of LASEs for application in humans will also require detailed studies in porcine models, studies in animal models of specific pathologies including slow‐healing wounds (e.g., in diabetes) and wounds that are susceptible to infection. To that end, future work will involve a comprehensive investigation into bioactives that can accelerate tissue repair following laser sealing and into delivery of effective antimicrobial drugs for combating infections. In all these studies, a comprehensive picture of functional, biomechanical, and histological performance of LASEs will be obtained in order to investigate the potential for translating this technology for clinical use.

## AUTHOR CONTRIBUTIONS

**Deepanjan Ghosh:** Conceptualization (equal); data curation (lead); formal analysis (lead); investigation (lead); methodology (lead); writing – original draft (lead); writing – review and editing (lead). **Christopher M. Salinas:** Data curation (equal); formal analysis (equal); methodology (equal); writing – original draft (equal); writing – review and editing (supporting). **Shubham Pallod:** Data curation (supporting); writing – review and editing (supporting). **Jordan Roberts:** Data curation (supporting); formal analysis (supporting). **Inder Raj S. Makin:** Data curation (equal); formal analysis (equal); investigation (equal); methodology (equal); resources (supporting); validation (equal); writing – review and editing (equal). **Jordan R. Yaron:** Data curation (lead); formal analysis (lead); methodology (lead); writing – original draft (equal); writing – review and editing (supporting). **Russell S. Witte:** Formal analysis (equal); investigation (equal); methodology (equal); resources (equal); validation (supporting); writing – review and editing (equal).

## CONFLICT OF INTEREST

Kaushal Rege is affiliated with a start‐up company, Synergyan, LLC. Other authors declare no conflict of interest.

### PEER REVIEW

The peer review history for this article is available at https://publons.com/publon/10.1002/btm2.10412.

## Supporting information


**Figure S1** In vivo live mouse imaging system. (A) GE Logiq *e* Nextgen ultrasound system equipped with a 10–22 MHz transducer. (B) Transducers used for imaging (i) 22 MHz transducer with 19.3 * 8.1 mm footprint and (ii) 18 MHz transducer with 34.8 * 11.1 mm footprint.
**Table S1.** Tissue processing steps for preparation of skin samples for histological and immunohistochemistry staining.

## Data Availability

The data that support the findings of this study are available from the corresponding author upon reasonable request.
