# Supplementary material for: Temporal evaluation of efficacy and quality of tissue repair upon laser‐activated sealing
Source: Bioeng Transl Med. 2022 Sep 28;8(2):e10412. doi: 10.1002/btm2.10412 (PMC10013809; doi:10.1002/btm2.10412)
Supplement: Supplementary file 1 — Figure S1 In vivo live mouse imaging system. (A) GE Logiq e Nextgen ultrasound system equipped with a 10–22 MHz transducer. (B) Transducers used for imaging (i) 22 MHz transducer with 19.3 * 8.1 mm footprint and (ii) 18 MHz transducer with 34.8 * 11.1 mm footprint. Table S1. Tissue processing steps for preparation of skin samples for histological and immunohistochemistry staining. [file BTM2-8-e10412-s001.docx]

**SUPPORTING INFORMATION**

**Temporal Evaluation of Efficacy and Quality of Tissue Repair upon Laser-activated Sealing**

Deepanjan Ghosh^a^, Christopher M. Salinas^b^, Shubham Pallod^a^, Jordan Roberts^c^, Inder Makin^d^, Jordan R. Yaron^a,e^ Russell S. Witte^b,f^, and Kaushal Rege^a,e#^

^a^ Biological Design, School for Engineering of Matter, Transport, and Energy

^c^ School of Life Sciences, Arizona State University, Tempe, AZ, 85287, USA

^e^ Chemical Engineering, School for Engineering of Matter, Transport, and Energy

Arizona State University, Tempe, AZ, 85287, USA

^b^ Optical Sciences

^f^ Medical Imaging

University of Arizona, Tucson, AZ, 85721, USA

^d^ School of Osteopathic Medicine, A.T. Still University, Mesa, AZ 85206, USA

^(#)^ To whom all correspondence must be addressed

Prof. Kaushal Rege

Email: rege@asu.edu


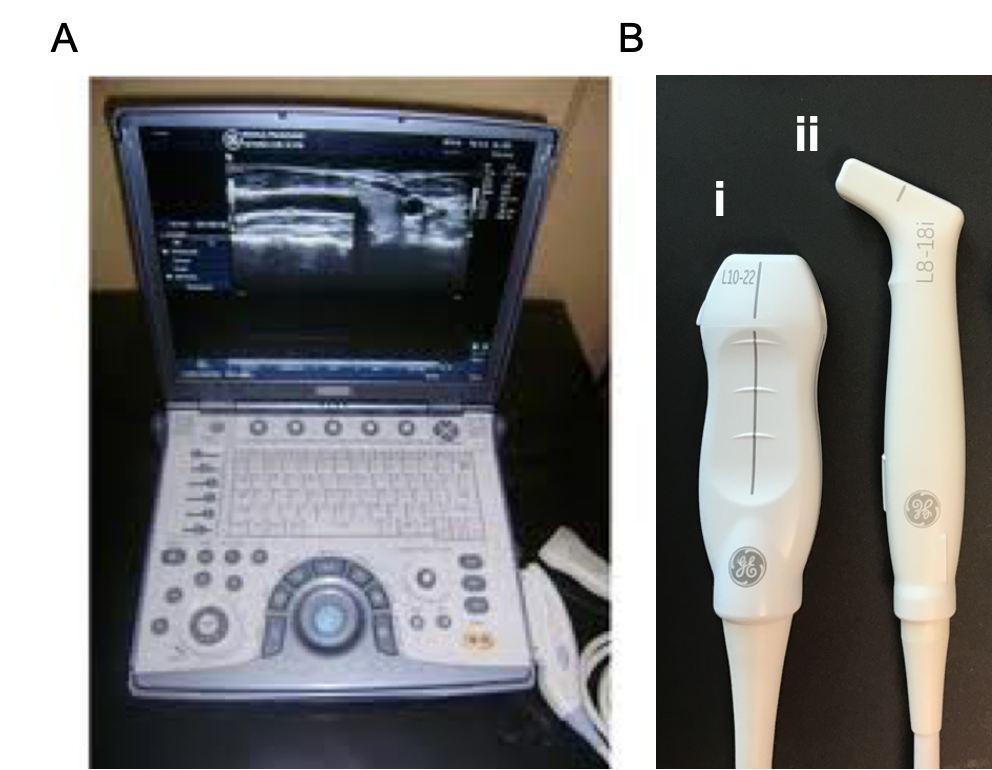


**Figure S1**. In vivo live mouse imaging system. (A) GE Logiq *e* Nextgen ultrasound system equipped with a 10-22 MHz transducer. (B) Transducers used for imaging (i) 22 MHz transducer with 19.3 * 8.1 mm footprint and (ii) 18 MHz transducer with 34.8 * 11.1 mm footprint.

| Step | Solution | Incubation time (in mins) | Temperature (in °C) |
| --- | --- | --- | --- |
| 1 | 1x PBS wash (to wash off excess formalin) | 15 | RT |
| 2 | 1x PBS wash (to wash off excess formalin) | 15 | RT |
| 3 | 40% Reagent Alcohol | 45 | RT |
| 4 | 70% Reagent Alcohol | 45 | RT |
| 5 | 70% Reagent Alcohol | 60 | RT |
| 6 | 90% Reagent Alcohol | 60 | RT |
| 7 | 90% Reagent Alcohol | 60 | RT |
| 8 | 100% Reagent Alcohol | 60 | RT |
| 9 | 100% Reagent Alcohol | 60 | RT |
| 10 | Xylene | 60 | RT |
| 11 | Xylene | 60 | RT |
| 12 | Xylene | 60 | RT |
| 13 | Xylene: Paraffin (1:1) | 30 | 60 °C |
| 14 | Paraffin | 45 | 60 °C |
| 15 | Paraffin | 45 | 60 °C |
| 16 | Paraffin | 45 | 60 °C |

**Table S1.** Tissue processing steps for preparation of skin samples for histological and immunohistochemistry staining.
